# Data on volatile compounds in fermented materials used for salmon fish sauce production

**DOI:** 10.1016/j.dib.2017.11.007

**Published:** 2017-11-07

**Authors:** Mitsutoshi Nakano, Yoshimasa Sagane, Ryosuke Koizumi, Yozo Nakazawa, Masao Yamazaki, Toshihiro Watanabe, Katsumi Takano, Hiroaki Sato

**Affiliations:** a Department of Food and Cosmetic Science, Faculty of Bioindustry, Tokyo University of Agriculture, 196 Yasaka, Abashiri, Hokkaido 099-2493, Japan; b The Organization for the Promotion of International Relationship, 709, 1-1-7 Motoakasaka, Minato-ku, Tokyo 107-0051, Japan; c Department of Applied Biology and Chemistry, Faculty of Applied Bioscience, Tokyo University of Agriculture, 1-1-1 Sakuragaoka, Setagaya-ku, Tokyo 156-8502, Japan

**Keywords:** Fish sauce, Volatile compound, Flavor, GC/MS

## Abstract

This article describes the analysis of volatile compounds in fermented materials used for salmon fish sauce production via gas chromatography/mass spectrometry (GC/MS). Ten types of fish sauces were produced from raw salmon materials, including various proportions of flesh, viscera, inedible portion (heads, fins, and backbones), and soft roe, by mixing them with salt and allowing them to ferment for up to three months. The volatile compounds were captured by a solid-phase microextraction method and then applied to GC/MS for separation and identification of the compounds in the fish sauce products. The number of volatile compounds identified in the starting materials varied from 15 to 29 depending on the ingredients. The number of compounds in the final fish sauce products was reduced by 3.4–94.7% of that in the original material. The retention times and names of the identified compounds, as well as their relative peak areas, are provided in a Microsoft Excel Worksheet.

**Specifications Table**

**Table:** 

Subject area	*Agricultural science*
More specific subject area	*Food chemistry*
Type of data	*Microsoft Excel Worksheet*
How data was acquired	*Volatile compounds in fermenting materials for salmon fish sauce production were analyzed using gas chromatography (GC: 7890A, Agilent Technologies, Inc.) coupled with mass spectrometry (MS: 5975C, Agilent Technologies, Inc.).*
Data format	*Analyzed*
Experimental factors	*Fish sauce was produced from raw salmon materials, which were purchased at a local market.*
Experimental features	*Determined the retention times and identified the volatile compounds and their relative peak areas from the GC chromatogram.*
Data source location	*Abashiri, Hokkaido, Japan*
Data accessibility	*Data are presented in a Microsoft Excel Worksheet, which is provided as supplementary data for this article.*

**Value of the data**

• The present data will help in understanding the origin of the characteristic flavor of salmon fish sauce products.
• The data will guide the design of manufacturing processes for salmon fish sauce products with superior flavor.
• The data will aid the discussion of the metabolism of fish sauce volatile components during the fermentation process.

## 1 Data

The Microsoft Excel Worksheet that is provided as supplementary data for this article lists the retention times and names of the identified volatile compounds in the fermenting materials used for salmon fish sauce production, as well as the relative peak area (in percentage) of each compound in the GC chromatogram.

## 2 Experimental design, materials, and methods

### 2.1 Design

Here, we employed two types of chum salmon (*Oncorhynchus keta*) raw materials for fish sauce production. Of the two, “Bunasake” is the fish after egg-laying, which is considered to have low marketable quality because of the loss of fat from the flesh [1]. In contrast, “Ginke”, which is the fish before egg-laying, is considered to have high marketable quality owing to the high content of fat in the flesh. Additionally, we employed *shio-koji*, a salt-marinated rice malt, to start the fermentation.

### 2.2 Materials

“*Bunasake”* salmon and “*Ginke”* salmon were purchased at a local market in Abashiri, Hokkaido. *Shio-koji* with 12.3% salt content (Kurashige Jozo Co. Ltd., Abashiri, Japan) and salt (Shokuen, The Salt Industry Center of Japan, Tokyo, Japan) were used for fish sauce production.

### 2.3 Fish sauce production

Raw salmon was dissected into flesh, viscera, inedible portion, and soft roe, and each portion was minced using a food processor. Each minced portion and salt were then mixed in various proportions, as shown in Table S1, and left for three months in a 37 °C constant-temperature incubator (DG-82, Yamato, Japan).

### 2.4 Volatile compound analysis

Solid-phase microextraction (SPME) was employed to quantify the compounds in the salmon fish sauce. Volatile compounds in the samples were extracted using a 2 cm, 23 gauge SPME fiber coated with 50/30 µm divinylbenzene/carboxen/polydimethylsiloxane (DVB/CAR/PDMS) (Supelco Co., Bellefonte, PA, USA). Then, 1 mL of fish sauce was placed in a 20 mL glass vial, and the samples were incubated at 30 °C for 5 min. After equilibration, the SPME fiber was exposed to the headspace for 30 min at the same agitation speed and temperature. The SPME fiber was then injected into a gas chromatograph at 250 °C for 5 min in splitless mode. The fermented materials were collected one, two, and three months after the initiation of fermentation and placed in vials with screw caps for SPME and analyzed by GC (7890 A, Agilent Technologies Inc., Santa Clara, CA. USA) using a DB-WAX column (30 m × 0.25 mm i.d., 0.25 µm film thickness; Agilent Technologies Inc.) coupled with MS (5975 C, Agilent Technologies, Inc.). The oven temperature was initially held at 60 °C for 3 min and then increased to 230 °C at the rate of 5 °C/min. Helium was used as the carrier gas at a flow rate of 1.75 mL/min. The compounds were identified based on their retention times in the GC column. The relative amounts of compounds in each processing step were determined using an extracted ion chromatogram from the MS analysis.
