# Supplementary material for: Data on volatile compounds in fermented materials used for salmon fish sauce production
Source: Data Brief. 2017 Nov 7;16:154–6. doi: 10.1016/j.dib.2017.11.007 (PMC5848111; doi:10.1016/j.dib.2017.11.007)
Supplement: Supplementary file 3 — Supplementary material [file mmc3.pdf]

Supplementary data 1. Results of GC/MS analysis of fermented materials for salmon fish sauce production. Retention times, names of the identified compounds, and relative peak areas (%) in the GC chromatogram are provided in the Microsoft Excel Worksheet.

Table S1. Proportions (%) of materials used for fish sauce production.

Product ID	Fish material	Flesh	Viscera	Inedible portion*	Soft roe	Salt	*"Shio-koji"*	Total
A	*"Bunasake"*	55.0	5.0	15.0	-	25.0	-	100
B		49.5	4.5	13.5	-	22.5	10.0	100
C		35.0	5.0	15.0	20.0	25.0	-	100
D		31.5	4.5	13.5	18.0	22.5	10.0	100
E	*"Ginke"*	55.0	5.0	15.0	-	25.0	-	100
F		49.5	4.5	13.5	-	22.5	10.0	100
G		35.0	5.0	15.0	20.0	25.0	-	100
H		31.5	4.5	13.5	18.0	22.5	10.0	100
I	Soft roe	-	-	-	75.0	25.0	-	100
J		-	-	-	67.5	22.5	10.0	100

*Inedible portion includes heads, backbones, and fins of fish.
